# Contribution of the Commensal Microflora to the Immunological Homeostasis and the Importance of Immune-Related Drug Development for Clinical Applications

**DOI:** 10.3390/ijms22168896

**Published:** 2021-08-18

**Authors:** Vanessa M. Beutgen, Carsten Schmelter, Norbert Pfeiffer, Franz H. Grus

**Affiliations:** Experimental and Translational Ophthalmology, Department of Ophthalmology, University Medical Center, 55131 Mainz, Germany; vbeutgen@eye-research.org (V.M.B.); cschmelter@eye-research.org (C.S.); norbert.pfeiffer@unimedizin-mainz.de (N.P.)

**Keywords:** gut microbiome, immune homeostasis, natural antibodies, immunomodulation, immunotherapy

## Abstract

Not long ago, self-reactive immune activity was considered as pathological trait. A paradigm shift has now led to the recognition of autoimmune processes as part of natural maintenance of molecular homeostasis. The immune system is assigned further roles beneath the defense against pathogenic organisms. Regarding the humoral immune system, the investigation of natural autoantibodies that are frequently found in healthy individuals has led to further hypotheses involving natural autoimmunity in other processes as the clearing of cellular debris or decrease in inflammatory processes. However, their role and origin have not been entirely clarified, but accumulating evidence links their formation to immune reactions against the gut microbiome. Antibodies targeting highly conserved proteins of the commensal microflora are suggested to show self-reactive properties, following the paradigm of the molecular mimicry. Here, we discuss recent findings, which demonstrate potential links of the commensal microflora to the immunological homeostasis and highlight the possible implications for various diseases. Furthermore, specific components of the immune system, especially antibodies, have become a focus of attention for the medical management of various diseases and provide attractive treatment options in the future. Nevertheless, the development and optimization of such macromolecules still represents a very time-consuming task, shifting the need to more medical agents with simple structural properties and low manufacturing costs. Synthesizing only the biologically active sites of antibodies has become of great interest for the pharmaceutical industry and offers a wide range of therapeutic application areas as it will be discussed in the present review article.

## 1. Introduction

The human gut microbiome (GM) consists of a plethora of different taxa [1]. The intestines are predominantly colonized with bacteria [2], but also several phyla of fungi [3], archaea [4] and viruses [5,6] were reported to be common components of the commensal microflora. The quantity and composition changes with the location within the intestine [7], but is also highly variable with age and geography [1]. A “healthy” or normal composition of GM has not been defined yet, but the Human Microbiome Project works on establishing commonalities that are valid for most parts of the population [8,9]. One of the major challenges in defining universal colonization patterns is that the gut microflora is highly individual, even in subjects living in the same environment [10,11].

For the study of GM composition, microbial species are commonly identified by analysis of fecal samples, mainly using 16s rRNA sequencing. However, distinct strains can only be identified with high-throughput, full-length 16s sequence data that can be obtained only recently with required accuracy [12]. Although this constitutes essential information, since different strains of the same species can evoke different effects for metabolism or the systemic host immune response. Another more laborious and expensive method is the shotgun metagenomics approach that can provide more detailed information about the microbial strains and displays a greater complexity of the GM [13].

Colonization with microbiota occurs after birth and mainly depends on mode of delivery and breastfeeding, but also other environmental traits are able to modify the composition of the microbiome [14]. In the first 2–3 years of life, the commensal microflora is unstable but is from there on, except for minor alterations, constant throughout adulthood [15]. However, major changes in the GM in adults can occur and are mainly promoted by dire changes in diet [16], lifestyle [17,18] or by antibiotic treatments [19].

The human GM plays a key role in the education and maintenance of the immune system, especially early in life [20,21]. The host immune system and the GM composition are in a circle of mutual control. Enteric microbiota shape the development of the immune system while the host immune response controls taxonomy and quantity of gut colonization. The nature of the intestinal microbiome has major influence on humoral and cellular immunity of the host. Beneath influencing the T cell responses, the composition of the B cell repertoire, and therewith the entirety of natural antibodies, is largely dependent on interactions with the commensal microflora [22,23]. Both, alterations in the GM and changes in the natural antibody repertoire are associated with different diseases [24,25,26,27,28,29].

Natural antibodies are defined as circulating immunoglobulins that are components of the physiological state of the organism and are present without prior immunization. IgM antibodies are most widely studied [30], but also antibodies of the classes IgG and IgA have been revealed as parts of natural immunity [31,32]. These antibodies show low affinity for potentially pathogenic organisms but also bind to self-antigens, which are referred to as natural autoantibodies. The origin and function of the natural (auto)antibody repertoire is still focus of ongoing research and several hypotheses developed [32,33].

Besides the importance to unravel the complex and versatile interplay between the GM and the host immune defense system, the implementation of immune-modulating therapeutic strategies has become of great interest for clinical applications. Particularly, commercial monoclonal antibodies are extremely valuable for the treatment of various disease pattern ranging from neurodegenerative and autoinflammatory disorders to highly aggressive and metastatic cancer types. In consideration of the tremendous progress in the development of medical treatment strategies, the trend is, nowadays, towards the manufacturing of small drug compounds with enhanced bioactivity instead of large biomolecules such as antibodies [34,35]. Focusing only on the active binding sites of antibodies, termed as complementarity-determining regions (CDRs), and their synthetic derivatives has become the center of attention for the medical management of various diseases and will offer completely new perspectives in the field of personalized translational medicine.

In the first part of the present review article, we want to highlight the connections between the GM and the natural antibody repertoire with special focus on systemic IgG, as well as their implications in various diseases. In the second part, we provide a comprehensive overview about current immunomodulatory treatment strategies by the exogenous supplementation of engineered therapeutic antibodies or their synthetic CDR peptides and discuss their highly specific and versatile modes of action.

## 2. Commensal Microbiome Shapes the Host Immune System

Immediately after birth, the neonate is exposed to a vast composition of microbes derived from the mother and the environment [36]. However, the prenatal and neonatal organism does not have a fully developed immune system and mainly depends on maternal antibodies to prevent enteric inflammation or other infections, which also can be mediated by the developing GM. Maternal Ig repertoires are delivered via the placenta or the colostrum and provide a first defense line against pathogens for the neonate. The colostrum contains maternal IgA, IgG and IgM antibodies that regulate gut microbiota and prevent bacterial translocation in the newborn [37,38]. The quality of the gut colonization in adults is primarily controlled through secretory IgA antibodies, but also anti-microbial IgG antibodies were shown to protect newborn mice from enteric and systemic *Escherichia coli* infections [38]. These protective antibodies belong to the maternal natural antibody repertoire [37,38].

The first 2–3 years of life are crucial for the development of a healthy immune system. Variation in colonization of different microbiota early in life can alter the host immunity and even increase susceptibility for autoimmune diseases and allergic reactions [20]. The GM plays a key role in this process as it constantly stimulates the host immune response and thereby educates the mucosal and systemic immunity. At the level of the epithelial gut cells that comprise the gut-vascular barrier, the immune system constantly interacts with commensal and potentially pathogenic microbiota and their metabolites. This border is the main contact point between microbiota and the host immune system and represents a training ground for the developing immunity. The interactions established here are necessary for the education and the correct development of an immune response that conveys a balance between tolerance and defense mechanisms.

A major task for the young host organism is the establishment of a tolerance to commensal, beneficial microbiota, while simultaneously educating the adaptive immune response against pathogens. Naïve T cells differentiate into effector T cells or regulatory T cells (Treg) upon contact to the gut bacteria. This depends on the type of bacteria and leads either to initiation of defense mechanisms or tolerance of the organism. Thus, the GM plays a pivotal role in the regulation of regulatory and proinflammatory T cell formation [39]. Treg cells are highly involved in the homeostasis of the GM and provide tolerance to commensal microbiota. A disturbance of the balance between immunosuppressive Treg cells and proinflammatory Th17 effector cells is associated with the susceptibility for autoimmune diseases [40]. The importance of this immune training by the gut microflora is shown in models of germ-free (GF) animals. Several studies revealed a deficiency of Th17 and Treg cells in GF mice that can be ameliorated by recolonization with common gut bacteria [41,42,43].

Immune tolerance in the gut, however, is not only established against microbiota, but also to food antigens. This priming of the immune system is referred to as oral tolerance. It mediates an active suppression of inflammatory processes against any antigens in the gastrointestinal tract, thereby preventing food allergies. There are two major ways in which oral tolerance can be promoted. On one hand, the induction of Tregs and on the other the clonal anergy of antigen-specific T cells. Both ways are not mutually exclusive and can both affect immune tolerance in a synergetic manner. Which of both mechanisms predominates is highly dependent on the amount of antigen that is administered orally. The mechanisms of oral tolerance were investigated as possible immunomodulatory treatment for various inflammatory and autoimmune diseases. Oral administration of antigens to induce tolerance and prevent an immune response is termed oral immune therapy (OIT). The efficacy of OIT has been investigated in the context of food allergies such as peanut allergy [44], but also ocular inflammatory diseases [45], type 1 diabetes [46], hemophilia [47] and others, some of which show promising effects in early clinical studies and/or animal studies.

The role of antibodies in the imparting of immune tolerance is not adequately investigated. It is currently known that humans show antigen-specific IgG, IgA and IgM to food antigens that show no apparent disease value [48]. Recent studies suggest a direct role of IgG in promotion of food tolerance by inhibition of IgE-mediated mast cell and basophil activation [49]. Especially, IgG4 is thought to promote maintenance of immunological tolerance [50]. This hypothesis is also supported by other studies showing an induction of food tolerance by maternal IgG from colostrum in the offspring [51,52].

The antibody-producing B cell repertoire as such is massively influenced by the GM and exhibits a circle of mutual control [53]. One part of this interaction is comprised by numerous IgA producing B cells in response to commensal bacteria [54]. The mucosal IgA controls the diversity of the GM. IgA-secretory B cells can be activated in a T cell independent way, but the Foxp3(+) T cell mediated activation appears to play a more pivotal role in GM homeostasis by regulating IgA selection at the Peyer’s Patches [55]. The influence of the commensal microflora on B cell diversity is not limited to the direct interaction sites in the gastrointestinal tract, comprising the mucosal immunity, but also effects systemic immunity [23]. Colonization of GF mice with different bacterial species elicited alterations in the B cell repertoire as measured by differentially secreted immunoglobulins. Biggest effects on B cell diversity were induced by systemic exposure of bacteria that could also naturally occur by bacterial translocation mediated by an impaired gut barrier. However, small effects on the IgG-secreting B cell pool were also observed with only mucosal exposure [23]. Other research groups likewise reported examples for the influence of the GM on systemic immunity. An earlier study of Zeng et al. [56] reported that a systemic IgG response can be triggered by gut bacteria under homeostatic conditions. Furthermore, commensal microbiota were shown to induce systemic IgA secretion in the serum of mice [54]. These findings suggest a role for the GM in forming and maintaining the systemic IgG antibody repertoire.

## 3. The Gut Microbiome Is Essential for the Homeostasis of the Natural Antibody Repertoire

**Figure 1:**
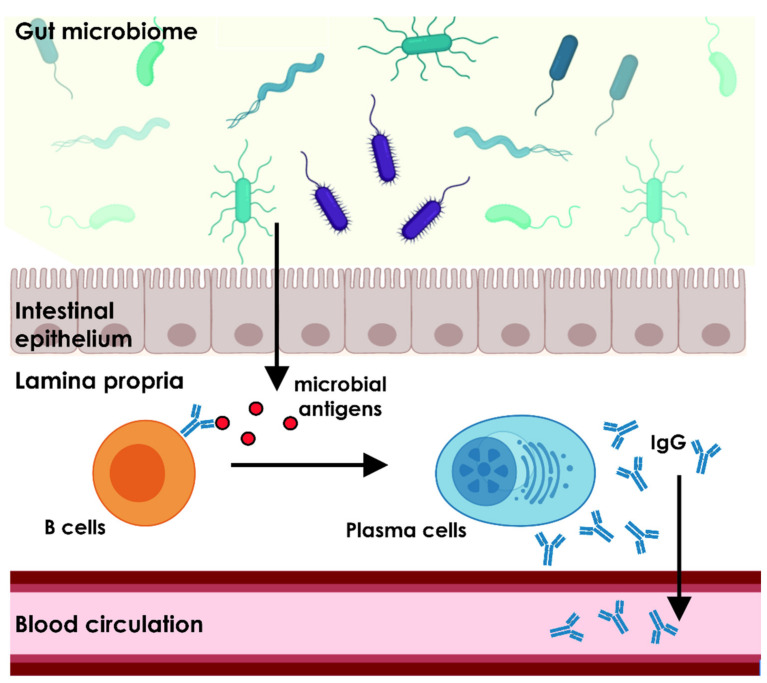
Putative influence of the commensal gut microbiome to systemic IgG-mediated immunity. Microbial antigens can pass through an impaired gut barrier, represented by the intestinal epithelium. The antigens bind to B cell receptors of B cells located in the lamina propria. Upon antigen recognition, the B cells differentiate into IgG secreting Plasma cells. The secreted immunoglobulins can enter the blood circulation or plasma cells directly translocate into blood vessels. Microbiota often exhibit highly conserved proteins with high sequence homology to the same proteins expressed in higher organisms including *homo sapiens*. This can lead to cross-reactivity of anti-microbial antibodies to self-antigens in the host organism via molecular mimicry. However, the gut microbiome has major influence on the diversity of the systemic antibody stock, regardless of their autoreactive potential. The figure was created with BioRender.com.

Natural antibodies are antibodies that are present at a physiological state of the organism without prior immunization to the antigen. They are hypothesized to be either spontaneously secreted by B cells or to be stimulated by intrinsic factors. Their existence and function are not undisputed among the immunology community. However, evidence supporting a key role for natural autoantibodies in the homeostasis of a healthy organism is accumulating. Here, we discuss possible connections between the origin of autoantibodies and the GM with special focus on the systemic IgG repertoire (Figure 1).

In humans, only IgG isotype antibodies can pass the placenta and can be transferred from the mother to the fetus via the neonatal Fc receptor (FcRn). Nevertheless, immunoglobulins of other classes, especially IgM, can be found in neonates [57]. Natural antibodies are further detectable in GF mice that were never exposed to an antigenic trait [58] and in bovine offspring that does not receive maternal immunoglobulins via the placenta [59]. It can be assumed that natural antibodies are secreted without external exposure to antigens and are formed either spontaneously or in reaction to intrinsic stimuli. An example for such a (semi-)internal trigger is the commensal microflora.

The natural antibody repertoire was found to be composed of immunoglobulins of the classes IgM, IgG and IgA. Natural antibodies are poly-reactive and bind their antigens with low affinity. They can bind to external antigens, as well as self-antigens. The main sources for these antibodies are B1 cells. B1 cells are a subset of B cells considered to be part of the innate immune response. Their main feature is their ability to spontaneously secret low-affinity antibodies, without prior antigen exposure [60]. This B cell subset was characterized in humans as cells with the marker profile CD20+ CD27+ CD43+ CD70- and can be further divided into CD5+ and CD5- B1 cells [61]. The B cell repertoire, in turn, is strongly shaped by the commensal microflora, including B1 cells [23,62]. With the B1 cell repertoire, also the natural antibody repertoire shows a high individual variation. Whether this is influenced by the likewise immense individual diversity of the GM is not yet sufficiently investigated.

The homeostasis of microbial and immune system interaction in the gut mainly depends on mucosal IgA secretion. However, the GM and its metabolites have not only local interactions with the host immunity at the epithelial gut barrier in the lamina propria, but they also have a major influence on systemic immunity. Several studies give evidence for the emergence of systemic IgG against intestinal microbial antigens. Their occurrence was first linked to impaired epithelial gut barrier function as observed in inflammatory bowel disease that lead to dislocation of gut bacteria [63]; but also more studies found serological IgG in healthy individuals indicating more far-reaching effects of the commensal microflora on systemic immunity [64,65].

Many central studies on the effects of the GM on the systemic natural antibody repertoire were using GF mice as model systems. GF mice show an overall impaired B cell development and are at a deficit of natural autoantibodies, while showing increased susceptibility for bacterial infections [66]. In an early study of Bos et al. [58], GF mice, fed with a defined ultra-filtered diet, showed only 5% of IgA and IgG levels in serum compared to a conventional mouse strain, while IgM levels were unchanged. This leads to the conclusion that IgM secreting plasma cells only undergo an IgA or IgG class-switch upon antigenic stimulation from the GM. This implies a crucial role of the GM for the development and maintenance of a general systemic antibody repertoire and especially shapes the diversity of the circulating IgA and IgG antibodies. Constant stimulus from the GM would also explain the persistent secretion of natural IgG antibodies that would be necessary to maintain constant levels of these immunoglobulins with a half-life of only 1–3 weeks, dependent on the respective IgG subclass [67,68]. Li et al. [23] further showed that mucosal microbial colonization of GF mice with a non-replicating *E. coli* strain was capable to elicit a weak serum IgG response. They also found that these serological antibodies bind to antigens expressed on the *E. coli* membrane, as well as cytosolic antigens. Among these cytosolic proteins, the serum IgG antibodies predominantly bind to ribosomal proteins. Systemic IgG was also found to mainly bind to bacteria of the phylum proteobacteria [65].

In the entirety of natural antibodies, also self-reactivity is a common characteristic. It is not unlikely to consider a cross-reactivity of anti-microbial antibodies and self-antigens, as many proteins are highly conserved and have homologues in higher vertebrates. For example, autoantibodies to heat shock proteins are frequently found in sera of diseased and healthy individuals [69,70]. This further supports the hypothesis that at least a part of the natural antibody repertoire, with emphasize on the IgG repertoire, can be attributed to the interaction of the host immune system and the commensal microflora.

The known immunoglobulin repertoire to commensal microbiota and the repertoire of natural antibodies exhibit some similarities. As shown for GM induced immunoglobulins [23], natural antibodies and autoantibodies also bind to intracellular antigens as well as membrane proteins [71]. Furthermore, IgG antibodies to commensal bacterial strains were shown to be poly-reactive and have a cross-reactivity to antigens from different species [72] and thereby share a common characteristic of natural autoantibodies [73]. Additionally, natural IgG autoantibodies in human sera were reported to show high intra-individual stability over time [31] while it was correspondingly shown that the IgG response to antigens originating in the commensal microflora remains stable during adulthood likewise [74]. Further, antibodies of the natural autoimmunity were found in mouse models to be generated by the T cell independent route by B1 cells, just like microbiota-binding antibodies [62].

The human GM is priming the host immune system for the production of polyreactive, low-affinity antibodies as integral component of the natural antibody repertoire that serves as first broad defense line against potential pathogens. It forms a part of the innate immune system before a highly specific adaptive immune response is established. Constant stimulus of the commensal microflora helps to maintain this steady-state antibody repertoire and therefore plays a key role in immune homeostasis. In fact, natural antibodies were shown to bind several microbial and viral antigens without prior immunization, supporting the hypothesis of a function as innate defense mechanism [75,76]. However, natural antibodies frequently show self-binding characteristics. The function of their binding to self-antigens is still mostly unexplored. Natural autoantibodies are discussed to be caused by the highly poly-reactive nature of the general natural antibody repertoire and are redeemed to be a mere side effect. Conversely, alterations in abundance of natural autoantibodies are often associated with different maladies.

## 4. Perturbations of the Gut Microbiome and Autoimmunity in Different Disorders

Changes in the GM have been linked to a manifold of diverse conditions, not only concerning gut health. These range from autoimmune diseases to neurodegenerative disorders and even show associations with different malignancies. A common cause for systemic diseases in association to the commensal microflora is the occurrence of dysbiosis or translocation of bacteria. Dysbiosis can be caused by changes in diet or usage of antibiotics, but also by pathogenic microorganisms, causing inflammation and alterations of the gut barrier [77]. This can not only cause disorders in the gut but also be a systemic trait by eliciting a systemic immune response. Induction of inflammatory processes in other organs and the favored occurrence of autoimmune disorders are a possible consequence of this. Especially the translocation of gut microbiota via an impaired gut barrier can evoke a strong immune response. A weakened intestinal barrier can be caused by intestinal inflammation [78], but also by extensive exercise [79] or drug abuse [80].

The quality of the gut colonization can be a surrogate for general health and disease state, which is also discussed for the natural antibody repertoire. Whether there is a general causality of intestine dysbiosis and alterations in the natural (auto)antibody repertoire needs to be addressed in future studies. Currently, it cannot be clearly determined, whether gut dysbiosis and inflammation with subsequent alterations in immune homeostasis have a direct influence in autoimmune disease development. The causes for the onset of autoimmune disorders remains unknown and the mechanisms leading to pathology are not finally clarified. The loss of immunological self-tolerance is suggested to be a result of a genetic predisposition in combination with various environmental traits. Among these environmental factors, the influence of infectious processes is the most widely studied. However, the influence of microbiota, especially in the gastrointestinal tract, has also gained much interest, as changes in the GM are correlated with disease onset and progression [81,82]. Aside from that, natural antibodies of the IgM class have been shown to possess protective effects against the onset of pathological autoimmune processes, as shown in mouse models, where depletion of IgM led to an increment of pathogenic IgG that resulted in lupus-like symptoms [57]. Direct causality remains to be proven, but some links between GM, the natural antibody repertoire and autoimmunity have been suggested in the study of several diseases in the autoimmune and neurodegenerative disorder spectrum, nevertheless. These findings are discussed in the following paragraphs.

A weakened or impaired mucosal gut barrier can lead to the emergence of autoimmune diseases caused by translocation of gut microbiota. This causes a systemic immune response to the translocated microbes by secretion of IgG antibodies. In some cases, autoimmune disorders can arise in this context, when a cross-reactivity of antibodies to microbial antigens and self-antigens with high target specificity occurs. This was recently supported by Ruff and colleagues [83], who reported cross-reactivity of an epitope of *Roseburia intestinalis*, a common gut colonist, and the autoepitope β2-glycoprotein I in antiphospholipid syndrome. This concept is known as molecular mimicry and is suggested to be one of the main reasons for the occurrence of autoimmune diseases. Molecular mimicry is based on similarity between microbial proteins and autoantigens. Here, sequence homologies in highly conserved proteins, e.g., the family of heat shock proteins, are especially prone to be affected by antibody cross-reactivity. However, mere systemic exposure to bacterial structures does not necessarily induce autoimmune processes, as host HLA-DR genotypes are crucial for autoimmunogenic potential of the microbial antigens [84].

Recent studies found that not only the translocation of gut microbiota is able to cause the onset of autoimmune disorders, but also the composition of the microbiome plays an essential role. A correlation between the quality of gut colonization and autoimmunity has been reported in various diseases including systemic lupus erythematosus (SLE) [85,86,87], rheumatoid arthritis [88] and Sjögren’s syndrome [89,90]. In these diseases, the composition of the GM was found significantly altered compared to healthy subjects. This, however, does not automatically imply a causation of the disease by an altered GM. Additionally, most studies investigate the interplay of the GM and autoimmunity in animal models and sophisticated clinical trials with human subjects are not extensively conducted. Even though, the GM has received a great deal of attention in the study of autoimmune diseases, the underlying pathomechanisms are still underexplored.

Neurodegenerative diseases are also impacted by an imbalance of gut microbiota and associated immune dysregulation. The central nervous system and the enteric nervous system are linked through communication via the gut-brain-axis [25,91]. Beyond this, the organisms of the GM are able to directly modulate the central nervous system [92]. With regard to neurodegenerative diseases, it has been argued that translocation of gut microbiota causes systemic inflammation able to impair the blood-brain-barrier, ultimately causing neuroinflammation, which in turn leads to neurodegeneration [25]. Neuroinflammation plays a major role in the most common neurodegenerative diseases Parkinson’s (PD) and Alzheimer’s disease (AD) [93]. In both disorders, alterations in the GM, together with changes in their metabolites, were observed and disturbance of the microbiota-gut-brain axis were identified as key factors contributing to neuroinflammation [94,95]. Apart from that, the IgG levels to some periodontal microbiota and *Heliobacter pylori* were found to be elevated in the serum or cerebrospinal fluid of AD patients [96,97,98]. There is further evidence showing a link between *H. pylori* infection and PD, also via related autoantibody secretion detected in sera of PD patients [99,100]. Levels of autoantibodies targeting antigens that have not been linked to microbiota were shown to be altered in these diseases as well and exhibit protective or pathogenic effects [101].

Studies in ophthalmology provide more evidence for the interplay of GM and immune system that drives pathology. Current investigations on this topic include studies of primary open-angle glaucoma (POAG), age-related macular degeneration and diabetic retinopathy [102]. Studies in rodents implicate a possible correlation of dysbiosis of the commensal microflora and the pathogenesis of these eye diseases [102]. Especially with glaucoma, the pathogenesis of another neurodegenerative disease is linked to alterations in the gut colonization. In POAG patients, gut colonization showed significant alterations for bacterial genera [103]. The quantity of *Megamonas* and *Bacteroides plebeius* was decreased, while *Escherichia coli*, *Prevotellaceae* and *Enterobacteriaceae* were found to be increased [103]. Another recent study by Chen et al. [104] showed that initial priming of the immune system by microbiota of the commensal microflora is necessary for an immune response in B and T cells causing glaucomatous neurodegeneration in the retina. They showed in a GF mouse model that the commensal microflora is necessary for the development of autoimmune T cells penetrating the retina and causing neurodegeneration. These T cells are primed by the GM and their receptors can bind to human and bacterial heat shock proteins.

The three disorders PD, AD and POAG share not only the commonality of altered GMs, patients also show alterations in their natural serological (auto)antibody repertoire, which have further been suggested as disease biomarkers [71,105,106]. Beyond the use as diagnostic tools, medical interventions, modulating the functionality of the natural antibody repertoire and exploitation of its properties, opens new treatment options for diseases of diverse spectra. Some natural antibodies, for instance, are found to a lesser extent in the context of some diseases. A suggested causality for this finding is the existence of a protective function of these antibodies. Decrement of potentially protective antibodies is often described in age-related diseases and is linked to an overall reduced quantity of natural antibodies due to declined numbers of B1 cells in the elderly [31,61,107]. This has been investigated in AD, where a loss of neuroprotective natural autoantibodies occurred with increasing age [108]. The supplementation of the decreased protective antibodies can have positive effects on neuron survival [109], and provides new treatment options. The utilization of such therapeutic antibodies is under investigation for the treatment of numerous diseases [110]. Thereby, not only the treatment with substituted antibodies is investigated but also synthetic antibody fragments such as CDR peptides are promising approaches to ameliorate the course of various disorders as will be discussed in more detail in the following chapter.

## 5. Immune-Related Drug Development in Clinical Applications

**Figure 2:**
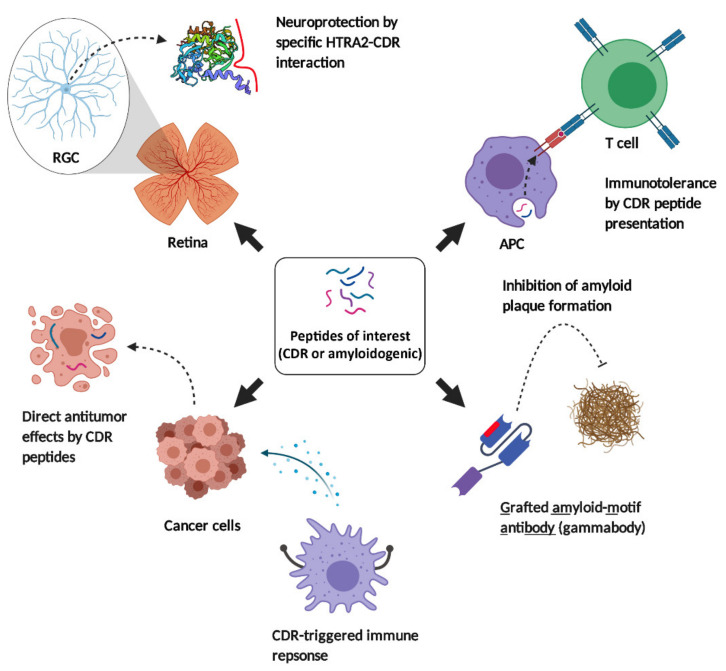
Different modes of action of synthetic CDR peptides and specifically engineered antibodies (gammabodies) for various therapeutic approaches. Synthetic CDR peptides are able to induce immunotolerance in SLE, neuroprotection in glaucoma and antitumor activities in melanoma. Specifically engineered antibodies in nanomolecluar size (gammabodies) are able to inhibit the formation of toxic amyloid plaques in Alzheimer’s and Parkinson’s disease. The abbreviation APC defines antigen-presenting cells. The figure was created with BioRender.com.

The development and engineering of synthetic immune-related pharmaceuticals, particularly therapeutic antibodies, is a rapidly growing market and offers great perspectives for the medical management of various diseases. Nevertheless, high manufacturing costs and large time investments are still the limiting factors for the large-scale production of such macromolecules and illustrate the urgent need of alternative strategies in pharmaceutical development. Humanization of antibodies by CDR grafting and phase-display technologies represent the gold standard for designing antibodies with high affinities for specific target molecules [110], but also possess several limitations and disadvantages (as summarized in [111]). Even single amino acid residues within grafted CDRs of humanized antibodies are known to show a certain degree of immunogenicity, which compromises their safety profile for clinical routine applications in humans. However, synthesizing only the biologically active site of antibodies has become of great interest as alternative therapeutic approach for various diseases and already delivered promising results in several study designs as well as clinical trials. Considering the trend towards the development of small drug compounds with enhanced bioactivity, these structural changes also improve the physicochemical properties of these molecules such as tissue penetration characteristics or resistance to enzymatic degradation. In the following sections the current application areas of synthetic CDR peptides and their different modes of action will be discussed in the autoaggressive disease SLE, the neurodegenerative disorders AD, PD and glaucoma as well as in cancer (Figure 2).

### 5.1. Systemic Lupus Erythematosus (SLE)

SLE is as a chronic autoimmune disease mainly characterized by a systemic dysfunction of B and T cells, increased production of autoantibodies (AAB) and the enhanced formation of immune complexes [112]. Patients suffering from SLE show a wide range of clinical manifestations ranging from mild symptoms such as joint paint or joint swelling to more serious effects such as heart failure or kidney dysfunction at more advanced stages of the disease [112]. Since long time it is already known that AABs with the specific idiotype 16/6 id (first identified in anti-ssDNA antibodies) show a high immunogenic potential and were able to enhance T cell-mediated immune responses in different SLE models [113,114]. AAB molecules containing the specific 16/6 idiotype are also increased in the serum of SLE patients and can induce an SLE-like phenotype by active immunization in BALB/c mice [112,115]. Interestingly, the subcutaneous injection of two synthetic CDR peptides (pCDR1 and pCDR3), encoding important sequence motifs of the pathogenic 16/6 idiotype antibody 5G12, were able to ameliorate the clinical symptoms of SLE in vivo and in vitro by triggering specific immunomodulatory activities [116,117]. Especially, the synthetic pCDR1 induced a lower expression of proinflammatory cytokines (INF-γ, TNF-α and IL2), a decreased T cell proliferation and also a decreased production of pathogenic AAB molecules in different SLE models [118,119,120] and/or in peripheral blood mononuclear cells (PBMC) of SLE patients [120]. On the other hand, the synthetic pCDR1 stimulated the up-regulation of immunosuppressive cytokine levels (e.g., TGF-β), increased the expansion of Tregs [118,120] and resulted in the decreased expression of MHCII complexes and costimulatory molecules on antigen-presenting cells (APCs) [121]. Furthermore, by creation of a peptide library of pCDR1 with single amino acid substitutions at specific positions (particularly D14 and S16), it was proven that the immunosuppressive properties of synthetic pCDR1 were diminished and failed to inhibit proliferative T cell responses [122]. It is assumed that the targeted amino acid substitutions interfere with the binding properties of pCDR1 to MHCII complexes on APCs, which conversely abrogates the proper recognition process by T cell receptors and fails to trigger immunotolerance against anti-DNA antibodies with the 16/6 idiotype in SLE [122]. Thus, it can be hypothesized that the beneficial immunoregulatory effects of pCDR1 are elicited in a sequence-specific manner by presentation to the host’s adaptive immune system.

Based on the previous preclinical data, the drug compound Edratide (an optimized pCDR1) developed by the company Teva Pharmaceutical Industries was tested in a human clinical trial phase II [123]. Edratide was administered subcutaneously weekly at doses of 0.5, 1.0 or 2.0 mg for 26 weeks in 340 SLE patients (inclusive placebo group). At the end, Edratide failed to pass the primary endpoints of the clinical phase II study, which were mainly defined by significant improvements in the ‘Systemic Lupus Erythematosus Disease Activity Index 2000′ (SLEDAI 2K) between drug and placebo groups. Thereby, the SLEDAI-2K score is comprised of 16 clinical observations and 8 laboratory measurements [124] and represents a gold standard to assess SLE disease activity [125]. Nevertheless, Edratide showed overall a good safety and tolerability profile in all three treated groups. Interestingly, a dose rate of 0.5 mg Edratide per week met at least a secondary outcome in a subgroup of SLE patients receiving low levels of steroids or no steroids at all (compared to placebo group). It should be noted that the secondary outcome was determined by BILAC scoring index [126], which is a questionnaire based on patient’s history, examination findings and laboratory results. However, based on these observations the authors concluded that the immunomodulatory effects of Edratide were potentially masked by steroids and should be revaluated in a clinical trial with longer terms and more strict clinical guidelines regarding the steroid administration to SLE patients. In addition, new findings also revealed beneficial effects of pCDR1 (Edratide) in PBMCs of Sjörgren’s syndrome patients [127], which might be also a target group for further clinical trials in the future.

### 5.2. Neurodegenerative Diseases

#### 5.2.1. Alzheimer’s and Parkinson’s Disease

Abnormal protein deposits are one of the common hallmarks in neurodegenerative disorders such as AD and PD and several new approaches have been developed to prevent the enhanced accumulation of these toxic protein aggregates in neuronal tissues such as the brain [128,129]. One of these new technologies was recently developed by the research group of Tessier, who grafted specific hydrophobic sequence motifs of amyloidogenic protein structures into the hypervariable CDR3 region of a single chain antibody scaffold (VH) [130,131,132]. Thereby, these specific hydrophobic peptides were derived from the Aβ protein (AD) as well as from α-synuclein (PD) and were termed as Grafted AMyloid-Motif AntiBODIES (Gammabodies) [130,131]. For the grafting process, they choose a highly stable and soluble variable antibody domain (VH), whose specific structural folding is insensitive for mutations in the CDR3 loop. Interestingly, the binding of the gammabodies to their misfolded amyloid targets is based on the recognition of the corresponding grafted peptide segments in the aggregates (homotypic interaction) and provides the removal of conformation- and sequence-specific protein deposits. Employing molecular dynamics simulations, a recent study [133] confirmed the importance of grafted CDR3 sequences for these specific homotypic interactions, while the CDR1/CDR2 loops are much lesser needed for the proper contacts. The gammabody Aβ33-42 (CDR3 motif: GLMVGGVVIA), for instance, targets Aβ monomers, soluble Aβ oligomers and Aβ fibrils, whereas the gammabody Aβ15-24 (CDR3 motif: QKLVFFAEDV) targets only insoluble Aβ fibrils and illustrates the importance of the sequence specificity for these interactions [131]. The highlighted parts of both sequences are supposed to be the major binding motif for the specific gammabody-Aβ monomer/oligomer/fibril interactions and are located in the C-terminus and, respectively, in the central region of the original Aβ protein. In addition, the authors also revealed that the amyloidogenic peptides without ’immobilization’ in an antibody scaffold are unable to bind the corresponding Aβ peptide fragments or Aβ conformers [130] and can be possibly traced back to the fact that these peptides preferential self-assemble, even at sub-stoichiometric concentrations [134]. However, also gammabodies are highly susceptible to aggregation within a few days at 25 °C or even a few minutes above 70 °C. Therefore, targeted insertion of negatively charged amino acids at the edges of the CDR3 (N- or C-terminus) can overcome this problem and increase the solubility of the gammabodies without interfering with the affinity and the binding specificities of the molecules [135,136].

The major advantage of gammabodies is that they are already biologically active at sub stoichiometric concentrations (1:10 gammabody: monomer; molar ratio) and can inhibit fibril formation and their cytotoxic effects even at the nanomolar scale [130,131]. In contrast to this, conformation- and sequence-specific antibodies, targeting the same amyloidogenic epitope, are much less effective to prevent fibrillization events at the same concentrations and illustrate the significance to use gammabodies for the therapeutic removal of toxic protein deposits in neurodegenerative diseases. Remarkably, gammabodies prevent the formation of amyloid aggregates by forming small complexes with Aβ monomers and Aβ fibril intermediates, which are unstructured, benign and nontoxic to neuronal cells [131]. Furthermore, Mark et al. [137] extended the application scope of this new technology by creating high-affinity antibody (gammabody) libraries using natural diversity mutagenesis for affinity maturation and provided a high-throughput platform for the development of optimized gammabody molecules targeting specific amyloid structures. In accordance with that, the authors Sormanni et al. [138,139] introduced a novel in silico-based methodology for the rational design of specific CDRs in gammabodies, which are able to target any selected epitope in any disordered protein or protein region.

However, so far the clinical application of gammabodies as therapeutic agent resulted in the amelioration of Aβ-induced cognitive defects in a high-fat-diet-induced obese rodent model for AD [140]. In conclusion, all previous findings clarify the great advantages and the broad range of application areas of gammabodies in the medical management of neurodegenerative amyloid-associated diseases. In addition, it will serve as innovative engineering platform in the future for the development of unique high-quality and high-affinity antibody molecules in nanomolecular size.

#### 5.2.2. Glaucoma

Glaucoma is a slowly progressive neurodegenerative disease characterized by the loss of retinal ganglion cells (RGCs) and their axons resulting in irreversible blindness of the patients. In a recent publication of our group it was proven that the synthetic CDR1 peptide (CDR1 motif: ASGYTFTNYGLSWVR) triggers neuroprotective effects on RGCs during glaucomatous-like conditions ex vivo and is caused by direct interaction with the target molecule High temperature requirement protein A2 (HTRA2) [141]. Interestingly, the target protein HTRA2 is reported to have two main contrary biological functions: (1) important key regulator of apoptosis [142]; and/or (2) essential role in neuronal cell survival and mitochondrial homeostasis [143]. In general, the protein HTRA2 acts as mitochondrial serine protease and promotes the degradation of various protein targets such as antiapoptotic components and/or damaged and misfolded protein structures during cellular stress responses. In its native state, the catalytic activity of HTRA2 is blocked by the C-terminal PDZ domain, whereas this suppression effect is abolished by the binding of highly specific peptide ligands to the PDZ domain binding motif [144]. These peptide ligands were identified as short, hydrophobic polypeptides, which were derived most probably from the C-terminus of aggregated protein structures [144]. However, comprehensive structural analysis of these polypeptides revealed the sequence YYF(V) as one high-affinity major binding motif of the PDZ domain [145,146] and can be also recognized as crucial sequence part in the synthetic CDR1 peptide (CDR1 motif: ASGYTFTNYGLSWVR). 

The newest findings of our group verified the inhibitory effect of synthetic CDR1 on the protease function of HTRA2 and is most likely caused by physical interaction with the PDZ domain [147]. Furthermore, HTRA2 specifically interacts with a wide range of proteins in the retina comprising important regulatory pathway mechanism ER to Golgi anterograde transport (e.g., AP3D1), protein localization to membrane (e.g., FLNA), aggrephagy (e.g., PSMC1) and the pyruvate metabolism/citric acid cycle (e.g., SHMT2) [147]. Thereby, the exogenous application of synthetic CDR1 peptide or protease inhibitor UCF-101 (blocking the catalytic domain of HTRA2) highly influenced the HTRA2-specific interactome in the retina and might highlight the importance of these signaling pathways in the pathogenesis of glaucoma. Nevertheless, how exactly the synthetic CDR1 peptide influences the biological functions of HTRA2 has to be addressed in future studies and will aid to unravel the complex neuroprotective mechanisms on RGCs in glaucoma.

In addition, the neuroprotective CDR1 sequence motif was revealed by quantitative mass spectrometry profiling of the highly diverse antibody repertoire in glaucoma patients [148] and is most likely shared between several systemic antibody molecules (or rather their respective B cell clones). Thus, this interesting observation may serve as excellent showcase for the complex interplay between the systemic humoral immune response and various external environmental stimuli such as the gut microbiome. In conclusion, all these new findings highlight the importance of innovative immunoproteomic profiling strategies for biomarker discovery and drug development and offer completely new perspectives for the medical management of various autoimmune-related diseases.

### 5.3. Cancer and Melanoma

Malignant and metastatic melanoma is one of the most aggressive forms of skin cancer and is responsible for 60–80% of lethal cases worldwide [149]. The malignant tumors derived from transformed melanocytes show a dysfunction in cell proliferation, apoptosis as well as disturbed cell-to-cell interactions [150,151]. This results in uncontrolled cell proliferation, tissue invasion and metastasis [152] and the 5-year survival of patients with metastatic, malignant melanoma is only predicted around 5–20% [149,150]. Additionally, drug resistance becomes an increasing problem in the medical therapy of melanoma [152] and illustrates the urgent need of new, innovative treatment strategies in cancer research. In the last decade, the research group of Polonelli and Travassos extensively proved that synthetic CDR peptides of different IgG and IgM monoclonal antibodies trigger various kinds of antimicrobial, antiviral, antifungal and antitumor activities in vitro/in vivo and are independent of the antigen specificities of the native antibodies [153,154,155,156,157]. One main objective of their current research work is the investigation of the molecular mechanism in metastatic and aggressive melanoma cells and the development of peptide-based immunotherapies to elicit effective and specific immune responses against malignant melanoma tumors. Recently, the research group engineered a series of synthetic CDR peptides, which were derived from several monoclonal antibody molecules, and evaluated their versatile antitumor characteristics in various in vitro and in vivo melanoma model systems. In the following sections, we will describe in detail the different modes of action of two synthetic CDR drug candidates and discuss their potential role for cancer immunotherapy in the future.

The synthetic peptide C36L1, which encodes the CDR1 from the variable light chain of an anti CD33 antibody, was identified first as bioactive molecule in a drug discovery screening for cancer therapy and showed cytotoxic effects against murine melanoma cells and a panel of human tumor cell lines in vitro [158]. Another study provided further evidence that C36L1 was actively taken up by melanoma cells and seemed to interact with microtubuli, causing depolymerization, endoplasmic reticulum stress and intrinsic apoptosis [159]. In addition, cell culture experiments revealed that C36L1 inhibits proliferation, migration and invasion of murine melanoma cells in vitro by cell cycle arrest at G2/M phase and by modulation of the PI3K/Akt signaling pathway [159]. It also delayed the tumor growth of subcutaneously grafted melanoma and their lung metastasis rates in mice in vivo [159]. Importantly, the authors Figueiredo et al. [160,161] identified the molecular target CD74 of synthetic C36L1 by using state-of-the-art molecular dynamic and binding prediction simulations and verified this specific interaction by dot-blot binding assay. Thereby, this in silico approach requires the crystal structure of the potential target protein (e.g., RCSB protein database) and the sequence of the assumed interaction candidate for binding prediction probability. This finding illustrates the power of computer-based simulations for molecular interaction elucidation, particularly for peptide-based immunotherapeutics. Molecular target CD74 is a highly expressed membrane receptor on the surface of dendritic cells (DC) and macrophages (MP) and shows a high affinity for the macrophage migration inhibitory factor (MIF), which is highly expressed by melanoma cells [162]. The MIF CD47 signaling pathway has important immunosuppressive functions such as anti-inflammatory cytokine release (e.g., TGF-β) and/or tolerogenic properties on DCs as well as MPs. Interfering the MIF CD47 signaling on the effector cells by peptide-induced receptor blocking can restore their antitumorgenic functions and, respectively, elicit effective immune responses against malignant metastatic melanoma [160,161]. Moreover, treatment with C36L1 also induced the proliferation of activated, cytotoxic T cells in metastatic lung in vivo, whereas the development of Tregs was diminished [160].

Another antitumor effector mechanism was elicited by synthetic cyclic peptide Rb9, which represents the VHCDR3 of the monoclonal antibody RebMab200 and original targets the NaPi2B phosphate-transport protein. Synthetic Rb9 induced hyper-adherent effects, suppression of cell migration and decreased matrix invasion in murine melanoma cells and in other human cancer cell lines in vitro [163]. Using molecular dynamics calculations, the authors Girola et al. [163] revealed that the cyclic arrangement of peptide Rb9, particularly stabilized by its central α-helix, leads to a more rigid structure compared to linear equivalent Rb10 and favors the orientation of the highly reactive cysteine residue (C92) towards nucleophilic structures in the target proteins or receptors. Based on this structural information, the authors verified the physical interaction of Rb9 with the C-terminal region (nucleophilic CC binding motif) of Hsp90 by disulfide bonds, which was abolished by the additional treatment with reducing agents. This specific interaction inhibited the anti-aggregating functions (chaperone function) of the target molecule Hsp90 and caused attenuation of the downstream FAK-Src-Rac1 signaling pathway [163], which is important for cancer migration and invasion. Enhanced expression of Hsp90 as cell surface receptor is known to facilitate cancer survival as well as progression and represents a favored target molecule in cancer immunotherapy (as summarized in [164]). However, Girola et al. [163] further confirmed an increased affinity of synthetic Rb9 for the C-rich GAIN domain of adhesion receptor GPR124, which is expressed on the cell surface of murine melanoma cells. In this case, the authors assumed that synthetic Rb9 activates the adhesive properties of GPR124 to different cell substrates such as extracellular matrix or cell monolayers resulting in inhibitory effects on tumor migration and invasion. In another recent study of the research group [165], it was shown that synthetic Rb9 is able to suppress melanoma tumor growth and metastasis in syngeneic wildtype mice in vivo, but failed to induce these effects in immuno-compromised animals (NOD/Scid/IL 2Rγnull). Molecular level investigations revealed that these antitumor protective effects were mainly regulated by DCs and CD4+ T helper cells in this in vivo cancer model; thereby, synthetic Rb9 elicited diverse and highly complex immunomodulatory functions in these immune cells depending on their specific functional state and degree on differentiation [165]. Furthermore, the authors verified a physical interaction of Rb9 with the previously described MIF ligand and the CD74 receptor, which is supposed to be the most relevant signaling pathway for these immune cell-mediated antitumor effects in vivo [165].

Besides that, the research group of Polonelli and Travassos also screened other synthetic CDR derived peptides such as AC-101 H3 [166] and Rb44 [167] for anti-tumorigenic activities with different mechanism of action and might be of interest for readers with special interest in melanoma research. In conclusion, CDR peptide-based immunotherapy provides promising perspectives for the medical treatment of cancer, particularly melanoma, and may form the basis for a synergistic combination therapy in the future.

## 6. Conclusions

The studies presented in this review emphasize the importance of the gut microbiome on the host’s immune system. Based on the gathered evidence, the commensal microflora is hypothesized as continuous trigger for the formation and maintenance of the natural antibody repertoire. Disturbances in the complex homeostasis between GM and the natural humoral immunity is associated with various diseases. The explicit connection of commensal microbiota and natural antibodies remains underexplored and this assumption, however, is for the most part based rather on correlation then causality. Therefore, there is an urgent need to address this important topic in future studies. Nonetheless, immunomodulatory features can be exploited for specific therapeutic purposes. The present review further summarizes the great advantages of synthetic CDR peptides and specifically engineered gammabodies for the medical treatment of various diseases ranging from neurodegeneration and autoinflammation to highly aggressive and metastatic cancer types. All listed publications illustrate the versatile medical application areas of these small and highly bioactive drug compounds and will certainly make a huge contribution towards the field of personalized translational medicine in the future.
